# Yeast Assimilable Nitrogen Concentrations Influence Yeast Gene Expression and Hydrogen Sulfide Production During Cider Fermentation

**DOI:** 10.3389/fmicb.2020.01264

**Published:** 2020-06-24

**Authors:** Yangbo Song, Patrick Gibney, Lailiang Cheng, Shuwen Liu, Gregory Peck

**Affiliations:** ^1^College of Enology, Northwest A&F University, Yangling, China; ^2^Horticulture Section, School of Integrative Plant Science, Cornell University, Ithaca, NY, United States; ^3^Department of Food Science, Cornell University, Ithaca, NY, United States

**Keywords:** cider, diammonium phosphate, hydrogen sulfide, *Saccharomyces cerevisiae*, transcriptomics, yeast assimilable nitrogen

## Abstract

The fermentation of apple juice into hard cider is a complex biochemical process that transforms sugars into alcohols by yeast, of which *Saccharomyces cerevisiae* is the most widely used species. Among many factors, hydrogen sulfide (H_2_S) production by yeast during cider fermentation is affected by yeast strain and yeast assimilable nitrogen (YAN) concentration in the apple juice. In this study, we investigated the regulatory mechanism of YAN concentration on *S. cerevisiae* H_2_S formation. Two *S. cerevisiae* strains, UCD522 (a H_2_S-producing strain) and UCD932 (a non-H_2_S-producing strain), were used to ferment apple juice that had Low, Intermediate, and High diammonium phosphate (DAP) supplementation. Cider samples were collected 24 and 72 h after yeast inoculation. Using RNA-Seq, differentially expressed genes (DEGs) identification and annotation, Gene Ontology (GO), and Kyoto Encyclopedia of Genes and Genomes (KEGG) pathway enrichment, we found that gene expression was dependent on yeast strain, fermentation duration, H_2_S formation, and the interaction of these three factors. For UCD522, under the three DAP treatments, a total of 30 specific GO terms were identified. Of the 18 identified KEGG pathways, “Sulfur metabolism,” “Glycine, serine and threonine metabolism,” and “Biosynthesis of amino acids” were significantly enriched. Both GO and KEGG analyses revealed that the “Sulfate Reduction Sequence (SRS) pathway” was significantly enriched. We also found a complex relationship between H_2_S production and stress response genes. For UCD522, we confirm that there is a non-linear relationship between YAN and H_2_S production, with the Low and Intermediate treatments having greater H_2_S production than the High treatment. By integrating results obtained through the transcriptomic analysis with yeast physiological data, we present a mechanistic view into the H_2_S production by yeast as a result of different concentrations of YAN during cider fermentation.

## Introduction

Hard cider, simply referred to as cider in this paper, is a fermented beverage made from apple (*Malus* × *domestica* Borkh.) juice. Commercial cider producers use techniques and yeast strains similar to industry standards for grape-based wines. Hydrogen sulfide, which smells like “rotten eggs” (sensory threshold >0.00041 mg L^–1^), is a common off-aroma compound formed by yeast (*Saccharomyces cerevisiae*) during cider fermentation. Many environmental factors, such as nitrogen, vitamin, and metal ion concentration, fermentation temperature, juice turbidity, soluble solids concentration, acidity, fungicides, and sulfite (SO_2_) additions, have been reported to affect H_2_S production (Spiropoulos et al., 2000; Peck et al., 2016; Boudreau et al., 2017b). In particular, H_2_S is often released when nitrogen concentration is deficient in apple juice (Giudici, 1994; Sadhu et al., 2014).

There are three main pathways through which H_2_S may be produced by *S. cerevisiae*: degradation of sulfur-containing amino acids, the reduction of elemental sulfur, and the reduction of sulfite or sulfate (Hansen et al., 1994; Jiranek et al., 1995; Noble et al., 2015). When yeast produce H_2_S during fermentation, it is most often a result of the sulfate reduction sequence (SRS) pathway. *S. cerevisiae* reduces sulfate or sulfite for the synthesis of the sulfur-containing amino acids methionine and cysteine and their derivatives (Linderholm et al., 2008). The SRS pathway is multifaceted and responsive to numerous regulatory inputs. Both cysteine and methionine are chemically reactive under reductive fermentation conditions, which induces H_2_S and other undesirable sulfur-containing compounds (Boudreau et al., 2017a). Sulfite reductase is responsible for reducing sulfite to sulfide and is regulated by general amino acid control, cysteine, and methionine (Mountain et al., 1991). Previous reports suggest that cysteine and its derivatives, rather than methionine, are the main end products that regulate the pathway activity (Hansen and Johannesen, 2000).

Nitrogen is a critical nutrient that affects yeast growth (Crépin et al., 2014), fermentation performance (Freese et al., 2011; Brice et al., 2014; Walker et al., 2014), and the development of organoleptic qualities in ciders (Rollero et al., 2015). In the fermented beverage industry, nitrogen available for yeast metabolism is referred to as yeast assimilable nitrogen (YAN). YAN is the combination of organic [also known as free amino nitrogen (FAN)] and inorganic [ammonia (NH_3_) and ammonium (NH_4_^+^)] available for *S. cerevisiae* to use during fermentation. Free amino acids are the main constituent of YAN in apple juice. Although apple juice nitrogen concentration and composition can vary greatly among cultivars, geographic locations, and management practices, YAN concentrations are often reported to be below 140 mg N L^–1^, a value considered the minimum recommended YAN concentration for fermentation of fruit juice by *S. cerevisiae* (Boudreau et al., 2017b; Ma et al., 2018). Low nitrogen levels in apple juice may cause sluggish or incomplete fermentations (i.e., when not all of the sugars are metabolized by the yeast) (Blateyron and Sablayrolles, 2001). Commercial cider producers often add exogenous inorganic nitrogen in the form of diammonium phosphate (DAP) to compensate for low YAN apple juice. Interestingly, yeast may produce increased levels of H_2_S as a response to both low and high YAN concentrations depending on the yeast strain (Spiropoulos et al., 2000).

There are almost 30 nitrogen-containing compounds that can be metabolized by yeast (Godard et al., 2007). Research is mixed as to the sequential order that yeast metabolize different nitrogenous compounds. Zhang et al. (2018) suggested that ammonium, glutamine, glutamate, and asparagine are preferred over urea, proline, and allantoin, but other authors have found different responses depending on the yeast species and/or fermentation condition (Gobert et al., 2017). Sequential nitrogen assimilation is likely the result of differential regulation of the permeases involved in the uptake of these molecules (Crépin et al., 2012). As a preferred nitrogen source, ammonium supports growth when other preferred sources, such as glutamate and glutamine, are exhausted (Ljungdahl and Daignan-Fornier, 2012).

Several studies have reported on the wide genetic diversity of *S. cerevisiae* strains from both natural (often found in association with plants, soils, insects, etc.) and human-associated biotypes (Steensels et al., 2014; Marsit and Dequin, 2015; Dujon and Louis, 2017; Peter et al., 2018). The selective pressure imposed by adapting to diverse habitats has created a large number of natural mutants that can be used in the study of biochemical and physiological processes (Landry et al., 2006; Greig and Leu, 2009; Hittinger et al., 2015). Furthermore, humans have manipulated yeast genetics to obtain strains with specific properties for bread baking and producing various fermented beverages, including the fermentation of grains and fruit juices (Mortimer, 2000). Since the *S. cerevisiae* genome has been sequenced, it is an ideal candidate for transcriptomic studies. However, as a wide array of genes impact sulfide formation and release, a fair amount of yeast strain variability exists, which makes it difficult to develop universal fermentation management strategies to reduce or eliminate H_2_S production in commercial operations.

In this study, we took advantage of yeast strains that produce opposing H_2_S levels. The objective of this study was to determine gene expression related to H_2_S production that occurs for two yeast strains (UCD522 and UCD932) under three YAN levels [Low (L = 86 mg L^–1^), Intermediate (I = 208 mg L^–1^), and High (H = 433 mg L^–1^)] after 24 and 72 h of cider fermentation. RNA-Seq was used to obtain sequence data representing the entire set of RNA molecules transcribed within each treatment. A second objective was to characterize gene expression differences between UCD522 and UCD932.

## Materials and Methods

### Microorganisms and Media

The yeast (*S. cerevisiae*) strains, UCD522 and UCD932, were obtained from the collection of the Department of Viticulture and Enology, University of California, Davis. UCD522 is a commercially available *S. cerevisiae* strain that is widely used in commercial wineries and cideries. UCD522 produces relatively high amounts of H_2_S during alcoholic fermentation (Spiropoulos et al., 2000). UCD932 is a vineyard isolate that produces no H_2_S due to a natural mutation in which threonine was substituted for lysine at position 662 in the *MET10* gene. This allele behaves in a dominant manner over the other quantitative trait loci leading to reduced sulfide formation. The *MET10* gene encodes a part of the enzyme responsible for the conversion of sulfite to sulfide. The mutation does not allow “leakage” of H_2_S produced in the sulfate reductase out of the cell (Linderholm et al., 2010). Prior to use, yeast cultures were stored in 50% (v/v) glycerol at −80°C. A yeast extract peptone dextrose starter culture consisting of 20 g glucose⋅L^–1^; 10 g yeast extract⋅L^–1^; 20 peptone g L^–1^ was incubated for 48 h at 28°C in an orbital shaker (200 rpm).

### Apple Juice

Apple juice was produced from a mixture of culinary and European cider apples grown at the Cornell University Agricultural Experiment Station Research Orchards (Ithaca, NY, United States) in 2016. Whole apples were milled using a rotary grinder drum fitted with carbide teeth and pressed using a hydraulic rack and cloth pressing system (OESCO, Inc., Conway, MA, United States). The pre-fermentation juice chemistry was: YAN = 63.7 mg L^–1^, soluble solids concentration = 12.95 °Brix; titratable acidity = 3.45 g L^–1^; pH 4.15; and total polyphenols (as measured by the Folin-Ciocalteu Assay) = 1.1 g gallic acid equivalents⋅L^–1^. The same base apple juice was used for all fermentations. Juice was stored at −20°C prior to fermentation.

### DAP Treatments, Cider Fermentation Conditions, and Hydrogen Sulfide Detection

DAP was added to the apple juice at 22.3, 144.3, and 369.3 mg⋅L^–1^ to create Low, Intermediate, and High treatments, respectively. All other fermentation conditions [e.g., temperature, physical agitation, and potassium metabisulfite (160 mg L^–1^) additions] were kept constant.

Two sets of fermentations were carried out in a temperature-controlled room set at 20°C using 250-ml flasks filled to 40% of their volume with juice. One set was used to monitor fermentation progression; the other was used to collect samples for RNA extraction. The starter yeast was then added to 100 ml of apple juice at a rate of 1 × 10^7^ cells⋅ml^–1^. Fermentations were conducted in triplicate for each yeast strain × DAP treatment.

Fermentation rate was measured by mass loss every 24 h. Fermentations were considered completed when there was less than 0.01 g day^–1^ mass loss. Residual sugars in fermented ciders were measured using a Megazyme (Bray, Ireland) Sucrose, D-Fructose, and D-Glucose kit in a 96-well microplate spectrophotometric method at λ 340 nm. Residual sugar concentrations were not different among the treatments.

During fermentation, H_2_S was quantified daily using lead acetate selective gas detector tubes (Komyo Kitagawa, Japan), as described elsewhere (Ugliano et al., 2011). The detector tubes were introduced into the flasks through stoppers. During fermentation, the CO_2_ produced by yeast metabolism forced H_2_S through the detector tube. Reaction of H_2_S with the chemicals contained in the tube resulted in the formation of a colored band. The length of the band is proportional to the amount of evolved H_2_S. Detector tubes were replaced when the band was near the end scale. Readings were taken every day through the course of fermentation to obtain a profile of H_2_S released during fermentation.

### Total RNA Extraction

Samples for RNA sequencing and metabolomic analyses were taken 24 and 72 h after yeast inoculation. One fermentation sample per experimental unit was taken (2 time points × 3 DAP treatments × 3 replications = 18 total samples for each yeast strain). Aliquots of 4 × 10^7^ cells (4 ml) were taken at each time point before centrifugation at 1,000 × *g* for 5 min at 4°C. Total RNA was immediately extracted using the Hot-Phenol method (Sung et al., 2003) and contaminating genomic DNA was removed using the RNase-Free DNase I (Invitrogen), according to the manufacturer’s protocols. RNA purity and concentration were assessed using a NanoDrop 2000 Spectrophotometer (Thermo Fisher Scientific, Waltham, MA, United States) and Qubit^®^ 2.0 spectrofluorometer (Life Technologies, Carlsbad, CA, United States), respectively. Extractions yielded more than 100 ng RNA μl^–1^ with absorption characteristics of 260/280 above 2.0 and 260/230 above 1.8. Samples were then stored at −80°C.

### RNA-Seq Library Preparation

RNA-Seq libraries were constructed using the NEBNext Poly(A) mRNA Magnetic mRNA Isolation Module (NEB, Ipswich, MA, United States). For library preparation, non-ribosomal, polyA-containing mRNAs were enriched using polyT oligonucleotides. RT-PCR was used to establish accurate cycling for barcode incorporation and final library amplification, avoiding over- or under-cycling libraries, which could skew transcript counts during sequencing. Library fragment size was assessed by fragment analysis. A 2 nM equimolar pool was prepared for each lane of the Illumina HiSeq 2500.

### RNA Sequencing Analysis

Raw RNA-Seq reads were processed using Trimmomatic to remove adaptor and low-quality sequences (Bolger et al., 2014). Reads shorter than 40 base pairs (bp) were discarded. RNA-Seq reads were then aligned to the ribosomal RNA database (Quast et al., 2012) using Bowtie (Langmead et al., 2009) and the mappable reads were discarded. The resulting high-quality cleaned reads were aligned to the S288C genome (Nijkamp et al., 2010) using HISAT (Kim et al., 2015), allowing up to three mismatches. Raw counts for each S288C gene were derived and normalized to reads per kilobase of exon model per million mapped reads (RPKM). The raw counts were also fed to the edgeR program to identify differentially expressed genes (DEGs). Raw *P* values were corrected for multiple testing errors using the false discovery rate (FDR) (Benjamini and Hochberg, 1995). DEGs were identified, with a fourfold change (log_2_ fold change >2) as the criteria for significant gene expression difference.

### Gene Ontology and KEGG Enrichment Analysis of DEGs

Gene Ontology (GO) enrichment analysis of DEGs was implemented using the GOseq R package, which corrects for gene length bias. The GOseq method is based on Wallenius non-central hyper-geometric distribution compared with ordinary hyper-geometric distribution, which calculates the probability of GO term enriched by differential expression genes more accurately (Young et al., 2010). GO terms (including cellular components, molecular function, and biological processes) with corrected *P* values of <0.001 were considered significantly enriched by DEGs. KOBAS software was used to examine the statistical enrichment of DEGS in the Kyoto Encyclopedia of Genes and Genomes (KEGG^1^) pathways (Mao et al., 2005; Kanehisa et al., 2007). Specific gene functions and biological pathways were annotated according to Saccharomyces Genome Database (SGD^2^).

### qPCR Verification for Transcriptomic Data

Total extracted RNA was transcribed to cDNA using the Applied Biosystems^TM^ High-Capacity cDNA Reverse Transcription Kit (Ambion Inc., Carlsbad, CA, United States) for reverse transcription. Real-time PCR was carried out with Bio-Rad iTaq^TM^ Universal SYBR Green Supermix on a BIO-RAD IQ5 (Bio-Rad, Berkeley, CA, United States) and calculation of relative expression was carried out following the protocols outlined in Pfaffl (2004). Primer sets for the targeted genes are listed in Table 1. Gene expression was assessed relative to expression of actin (*ACT1*).

**TABLE 1:** Primers used to analyze two yeast strains (UCD932 and UCD522) under three diammonium phosphate (DAP) treatments [Low (22.3 mg L^–1^), Intermediate (144.3 mg L^–1^), and High (369.3 mg L^–1^)] in a cider fermentation.

Systematic name	Gene	Name	Base sequence
YNR058W	*BIO3*	BIO3F	TGTTTCCCTTGGTACCCTTATTCGT
		BIO3R	AAGATCTGTGCTGTGATTTTGGAGC
YNL076W	*MKS1*	MKS1F	TTTTAACTCGGCCAATGACATCACC
		MKS1R	AATTGTCTGTTTGGAGCAACGTCAT
YNL183C	*NPR1*	NPR1F	TTTCTGCCAATTCCAATGGTCCTTC
		NPR1R	ATAGGAACCAGTTGGCTGTCTTGAT
YJL110C	*GZF3*	GZF3F	AACGGAGTCTAAGGAAAGAAGCGAT
		GZF3R	GGGCAGTTTAGGCTTTAAGTTTGGT
YFR030W	*MET10*	MET10F	CAACGTCAGAGTGCCATTACCTTTT
		MET10R	GAAAGAAGGCGAAGAACCATCCAAA
YBR294W	*SUL1*	SUL1F	CTACCCTTTGTTTGCTTTGTGGGAT
		SUL1R	GAAGCCAGCAACAGCATTTAGAGAA
YGR155W	*CYS4*	CYS4F	TCGACTTAGTTGGTAACACCCCATT
		CYS4R	TGGCAATTCTGTCTTTGATGGAACC
YER091C	*MET6*	MET6F	CGCCATTGGTAACAAACAAACCTTG
		MET6R	CTCTGTCAGCACCCAACTTTTCAAT
YKL218C	*SRY1*	SRY1F	CGATGGACAACAATCCTTCAGATCG
		SRY1R	CAAATGTGTACTCACCGAGGTGTTG
YFL039C	*ACT1*	ACT1F	CAAGGCTGTAGAATTCGCTCAACAA
		ACT1R	TGTTCAAAACATCAGAGCCTTCGTC

### Statistical Analysis

Fermentation and H_2_S data were tested for normalcy and then subjected to a one-way analysis of variance (ANOVA) followed by mean separation with Tukey’s Honestly Significant Difference using JMP 5.0.1 (SAS, Cary, NC, United States). Linear regressions between RNA-Seq and qPCR results were carried out using R studio 3.4.4 (R Studio, Boston, MA, United States).

## Results

### Fermentation Characteristics

**FIGURE 1:**
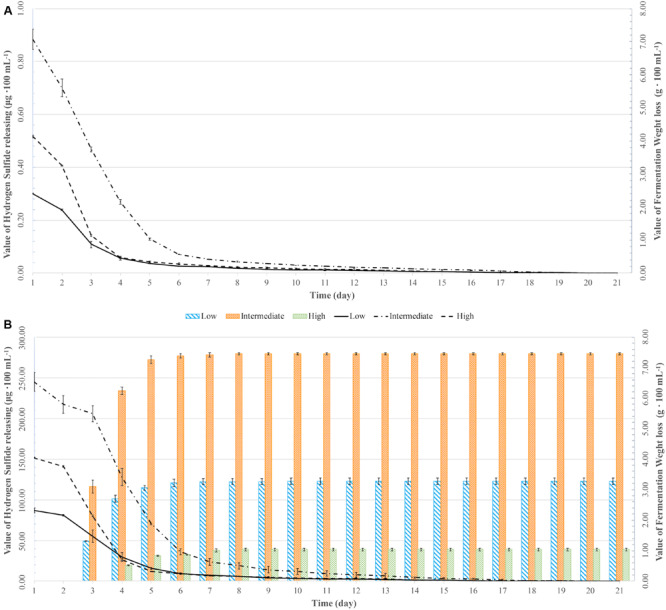
Weight loss over time (lines) and hydrogen sulfide (H_2_S) production (bars) for two yeast strains [UCD932 **(A)** and UCD522 **(B)**] under three diammonium phosphate (DAP) treatments [Low (22.3 mg L^–1^), Intermediate (144.3 mg L^–1^), and High (369.3 mg L^–1^)] in cider fermentations. Values reported as mean (*n* = 3) values ± SEM.

All fermentations lasted for at least 102 h (Table 2 and Figure 1). The longest fermentation duration was observed in the Intermediate DAP treatment; 174 and 222 h for UCD932 and UCD522, respectively. The Intermediate treatment resulted in a longer fermentation duration than the Low or High DAP treatments. The fermentation rate of both the Intermediate and High treatments was faster than the Low treatment. Mass loss of the two strains under three treatments were similar (*P* value = 0.00513). However, the fermentation profiles of the two strains diverged substantially at the 72-h time point when UCD932 under the Intermediate DAP treatment exhibited a higher fermentation rate (1.85 g day^–1^) than UCD522 (0.36 g day^–1^). Overall, the correlation between DAP concentration and fermentation kinetics was poor.

**TABLE 2:** Mean fermentation rate, fermentation duration, and mass loss production for two yeast strains (UCD932 and UCD522) under three diammonium phosphate (DAP) treatments [Low (22.3 mg L^–1^), Intermediate (144.3 mg L^–1^), and High (369.3 mg L^–1^)] in a cider fermentation.

Strain	DAP treatment	Fermentation rate (g day^–1^ 100 ml^–1^)	Fermentation duration (h)	Mass loss (g 100 ml^–1^)
UCD932	Low	0.02 ± 0.004 c	120.000 ± 19.596 cd	2.408 ± 0.015 c
UCD932	Intermediate	0.039 ± 0.004 a	174.000 ± 12.000 b	6.815 ± 0.57 a
UCD932	High	0.033 ± 0.003 ab	126.000 ± 12.000 cd	4.355 ± 0.512 b
UCD522	Low	0.016 ± 0.000 c	144.000 ± 0.000 c	2.318 ± 0.019 c
UCD522	Intermediate	0.029 ± 0.003 b	222.000 ± 12.000 a	6.320 ± 0.497 a
UCD522	High	0.040 ± 0.005 a	102.000 ± 12.000 d	4.058 ± 0.039 b

*Values expressed as the mean (*n* = 3) ± SEM. Values marked with different lowercase letters within each column are significant at *P* ≤ 0.05.*

### Hydrogen Sulfide Production

Hydrogen sulfide was not detected for the fermentations using the UCD932 yeast strain, regardless of DAP treatment concentration (Table 3 and Figure 1). For UCD522, the release of H_2_S started after 24 h for the High treatment and after 72 h for the Low and Intermediate treatments. Hydrogen sulfide production ceased after 192 h for all three UCD522 fermentations. Under the Low treatment, there was a positive correlation between the fermentation rate and the H_2_S production rate (*P* value ≤0.001; Pearson *R*^2^ = 0.95). In the UCD522 Intermediate DAP fermentation, H_2_S production was two and six times greater (288.250 μg 100 ml^–1^) than the Low (123.750 μg 100 ml^–1^) and High (44.125 μg 100 ml^–1^) treatments. Under the High DAP treatment, UCD522 produced the least amount of H_2_S throughout the fermentation.

**TABLE 3:** Mean hydrogen sulfide (H_2_S) production rate, production duration, and total H_2_S production for two yeast strains (UCD932 and UCD522) under three diammonium phosphate (DAP) treatments [Low (22.3 mg L^–1^), Intermediate (144.3 mg L^–1^), and High (369.3 mg L^–1^)] in a cider fermentation.

Strain	DAP treatment	H_2_S production rate (μg day^–1^)	H_2_S production duration (h)	Total H_2_S (μg 100 ml^–1^)
UCD932	Low	0 ± 0 d	0 ± 0 c	0 ± 0 d
UCD932	Intermediate	0 ± 0 d	0 ± 0 c	0 ± 0 d
UCD932	High	0 ± 0 d	0 ± 0 c	0 ± 0 d
UCD522	Low	1.323 ± 0.270 b	96.000 ± 19.596 b	123.750 ± 3.948 b
UCD522	Intermediate	2.196 ± 0.185 a	132.000 ± 13.856 a	288.250 ± 17.877 a
UCD522	High	0.349 ± 0.052 c	120.000 ± 0.000 a	44.125 ± 5.483 c

*Values expressed as the mean (*n* = 3) ± SEM. Values marked with different lowercase letters within each column are significant at *P* ≤ 0.05.*

### Differential Gene Expression

**FIGURE 2:**
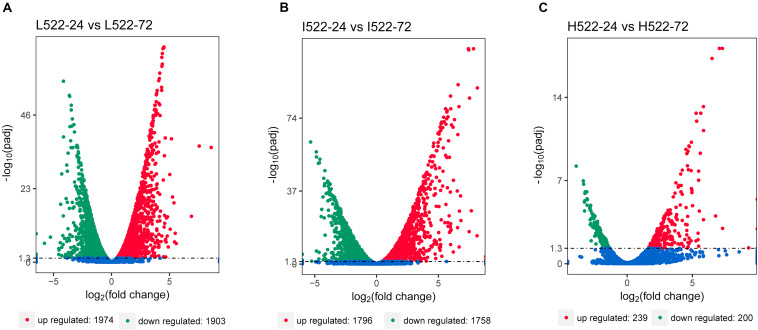
Volcano map of differentially expressed Saccharomyces cerevisiae (UCD522) genes under three diammonium phosphate (DAP) treatments [**(A)** Low (22.3 mg L^−1^), **(B)** Intermediate (144.3 mg L^−1^), and **(C)** High (369.3 mg L^−1^)] after 24 and 72 h in a cider fermentation. DEGs are shown as a red (up) or green (down) dot. Blue dots represent genes that were not different between the time points. Abscissa represents multiple genes expressed in different samples. Ordinate values on the axes represent the magnitude of gene expression change.

**FIGURE 3:**
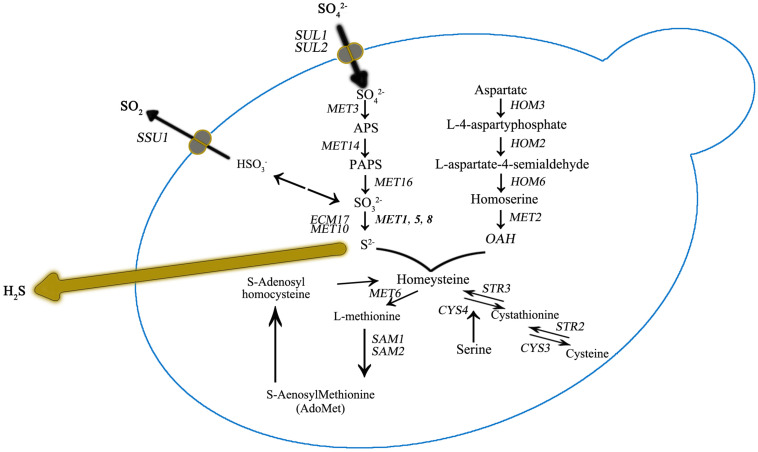
Sulfur amino acid biochemical pathways in *Saccharomyces cerevisiae*. Genes that encode for catalyzing enzymes are shown in italic.

Differential gene expression utilizing RNA-seq was used to quantify differences among the yeast strains (UCD932 = 932; UCD522 = 522), sample time points (24 = 24 h; 72 = 72 h), and DAP treatments (L = low; I = intermediate; H = high). The RNA-seq results were validated using qRT-PCR. Relative expression levels of selected genes between the methods were well correlated (*r*^2^ = 0.88; *P* ≤ 0.0001) (Supplementary Figure S1). Thousands of genes were differentially expressed between 24 and 72 h, under the three DAP treatments (Figure 2). For UCD522, there were 3,877 genes differentially expressed between L522-24 and L522-72 (1974 DEGs were up-regulated and 1,903 DEGs were down-regulated), 3554 genes differentially expressed between I522-24 and I522-72 (1796 DEGs were up-regulated and 1,758 DEGs were down-regulated), whereas 439 genes differentially expressed between H522-24 and H522-72 (239 DEGs were up-regulated and 200 DEGs were down-regulated) (Figure 3). Detailed DEGs for these three comparisons are provided in Supplementary Table S1.

For UCD932, between 24 and 72 h (L932-24 and L932-72) there were 826 DEGs (447 were up-regulated and 379 were down-regulated). Comparison of I932-24 and I932-72 was 4,615 DEGs (2332 DEGs were up-regulated and 2,293 were down-regulated). For H932-24 and H932-72, there were a total of 3,020 DEGs (1535 DEGs were up-regulated and 1,485 were down-regulated). Detailed DEGs for these three comparisons are provided in Supplementary Table S2.

It was also observed that the number of DEGs decreased as the DAP concentration increased (Figure 2). We further focused DEG statistical analyses on the SRS pathway for each comparison (Supplementary Table S1). We found that the expression level of *MET10* and *MET5* genes was impacted by the DAP treatments (Figure 3). Most of the genes of the SRS pathway were highly expressed at the 24-h time point when no H_2_S was detected and down-regulated in the later fermentation stages, which coincided with H_2_S production.

**FIGURE 4:**
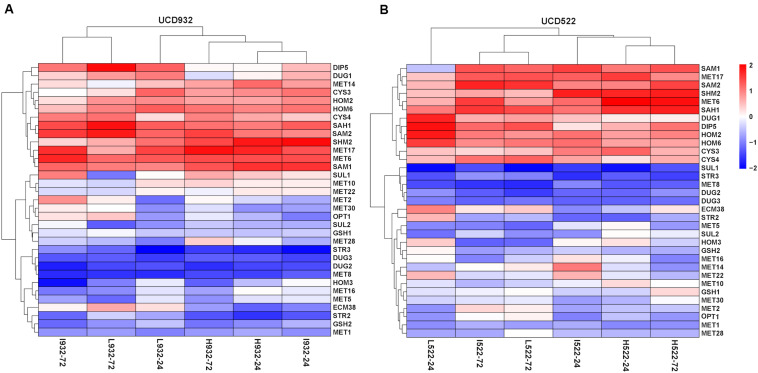
Heatmap of expression levels of sulfate reduction sequence core genes in two strains. Expression level (FPKM) for 33 genes encoding the core enzymes for sulfate reduction sequence for UCD932 **(A)** and UCD522 **(B)** under three diammonium phosphate (DAP) treatments [Low (22.3 mg L^–1^), Intermediate (144.3 mg L^–1^), and High (369.3 mg L^–1^)] after 24 and 72 h in a cider fermentation.

For both yeast strains, the Low and Intermediate DAP treatments had similar hierarchical clustering of the SRS gene expression, at 72 h (Figure 4). Under all treatments, *CYS4*, *HOM2*, *HOM6*, *MET6*, *MET17*, *SAH1*, *SAM2*, and *SHM2* were consistently up-regulated, only *CYS3* was up-regulated in UCD522, and only *SAM1* was up-regulated in UCD932. Conversely, under all treatments, *DUG2*, *DUG3*, *MET1*, *MET8*, and *STR3* were consistently down-regulated. Only *SUL1* was down-regulated in UCD522, while *GSH2*, *MET5*, and *STR2* were all down-regulated in UCD932. Under the treatments that produced more H_2_S (Low and Intermediate), UCD522 had a down-regulated *MET10* gene expression. *MET10* was up-regulated by UCD932, particularly at the 24-h time point, in all three DAP treatments. Several SRS genes showed reverse direction regulation between the two yeast strains under Low (24 h: *MET14*, *SAM1*, *SUL1*, and *HOM3*), Intermediate (24 h: *MET16* and *SUL1*; 72 h: *SUL1*), and High (24 h: *MET14*, *MET22*, *SUL1*, and *HOM3*; 72 h: *GSH2*, *MET1*, *SUL1*, *ECM38*, and *GSH1*) treatments. For UCD522, the comparison of SRS gene expression between the Low and High conditions, *ECM38*, *MET22*, and *MET10*, had reverse direction regulation at the 24-h sampling point, while *MET2* and *OPT1* also had reverse direction regulation at 72 h (Table 4).

**TABLE 4:** Genes affecting hydrogen sulfide production in *Saccharomyces.*

Gene name	Function^1^
*MET1*	*S*-Adenosyl-L-methionine uroporphyrinogen III transmethylase
*MET2*	L-Homoserine-*O*-acetyltransferase
*MET 5, 10*	Sulfite reductase beta subunit
*MET6*	Cobalamin-independent methionine synthase
*MET8*	Bifunctional dehydrogenase and ferrochelatase
*MET14*	Adenylylsulfate kinase
*MET17*	*O*-Acetyl homoserine-*O*-acetyl serine sulfhydrylase
*MET22*	Bisphosphate-3’-nucleotidase
*CYS3*	Cystathionine gamma-lyase
*CYS4*	Cystathionine beta-synthase
*SAH1*	*S*-Adenosyl-L-homocysteine hydrolase
*SAM1*	*S*-Adenosylmethionine synthetase
*SAM2*	*S*-Adenosylmethionine synthetase
*SHM2*	Cytosolic serine hydroxymethyltransferase
*DUG2*	Component of glutamine amidotransferase (GATase II)
*DUG3*	Component of glutamine amidotransferase (GATase II)
*STR2*	Cystathionine gamma-synthase, converts cysteine into cystathionine
*STR3*	Peroxisomal cystathionine beta-lyase
*SUL1*	High affinity sulfate permease of the SulP anion transporter family
*GSH1*	Gamma glutamylcysteine synthetase
*GSH2*	Glutathione synthetase
*ECM38*	Gamma-glutamyltranspeptidase
*OPT1*	Proton-coupled oligopeptide transporter of the plasma membrane
*HOM2*	1-Aspartic beta semi-aldehyde dehydrogenase
*HOM6*	Homoserine dehydrogenase

*^1^Function obtained from Saccharomyces Genome Database (http://www.yeastgenome.org/).*

For UCD522, after 24 h, the expression quantity of *HOM3*, *MET10*, *MET2*, and *SAH1* genes had a positive correlation with the increasing DAP concentration, and *HOM2* had a negative correlation. Furthermore, for the samples of UCD522 that were acquired during the liberation of H_2_S (at 72 h), there were negative correlations between the expression level of *CYS3*, *GSH1*, and *MET10* and the H_2_S concentration. However, this metabolic pathway also requires the two regulatory subunits encoded by *MET1* and *MET8*, and we did not find any correlation with the expression levels for those genes. Our data suggest that the genes that impact H_2_S production in a cider fermentation are under a tight regulatory control both during biosynthesis (*MET5* and *MET10*) and sulfide incorporation (*MET17* and *MET2*) (Table 4).

### Gene Expression Levels and Sulfur Compound Production

Under the Low and High DAP treatments, the *MET17* gene, which encodes the bifunctional *O*-acetylserine/*O*-acetylhomoserine sulfhydrylase (OAS/OAH SHLase), was both up- and down-regulated, but the production of H_2_S was only minimally affected. At the 24-h sampling point, under the Intermediate treatment, the expression of *MET17* was nearly eightfold greater than the Low treatment, but at 72 h, there was more H_2_S production. At the same time point, expression of *MET17* resulted in a 10-fold increase over the low one in OAS/OAH SHLase activity in UCD522, but had no impact on the level of H_2_S produced. Thus, transcriptome activity and H_2_S production were not well correlated in our study.

Most of the genes of the SRS pathway were highly expressed at the beginning of fermentation, when no H_2_S could be detected, and down-regulated in the later stages, which coincided with H_2_S production (Figure 4). The higher expression of SRS genes in the fermentation did not correlate with the lower levels of H_2_S production observed.

### GO Enrichment of DEGs Between UCD522 and UCD932

To further understand the function of the DEGs underlying the effect of concentration of YAN on the two yeast strains, GO enrichment analysis was performed with the DEGs of six comparisons (L522-24 vs L522-72, I522-24 vs I522-72, H522-24 vs H522-72, L932-24 vs L932-72, I932-24 vs I932-72, and H932-24 vs H932-72). DEGs were assigned to one or more GO terms and categorized into 1,770 (L522-24 vs L522-72), 1800 (I522-24 vs I522-72), 834 (H522-24 vs H522-72), 1128 (L932-24 vs L932-72), 1923 (I932-24 vs I932-72), and 1,664 (H932-24 vs H932-72) GO terms in the three main categories (biological process, molecular function, and cellular component) (Supplementary Table S3).

**FIGURE 5:**
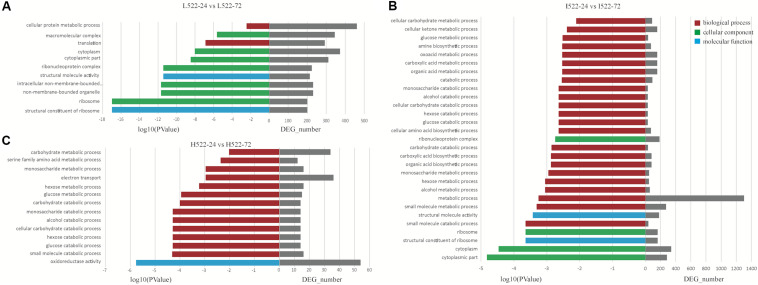
Enriched (P value < 0.001) Gene Ontology (GO) terms associated with Saccharomyces cerevisiae (UCD522) genes under three diammonium phosphate (DAP) treatments [**(A)** Low (22.3 mg L^−1^), **(B)** Intermediate (144.3 mg L^−1^), and **(C)** High (369.3 mg L^−1^)] after 24 and 72 h in a cider fermentation.

For UCD522, under the High treatment, nine GO terms were enriched in the category of biological process, including “small molecule catabolic process,” “glucose catabolic process,” “hexose catabolic process,” “cellular carbohydrate catabolic process,” “alcohol catabolic process,” “monosaccharide catabolic process,” “carbohydrate catabolic process,” “glucose metabolic process,” and “hexose metabolic process” (Figure 5). In the category of molecular function, “oxidoreductase activity” was significantly enriched.

For the Intermediate treatment, in the category of biological process, “small molecule metabolic process,” “metabolic process,” “alcohol metabolic process,” “hexose metabolic process,” and “small molecule catabolic process” were significantly enriched (Figure 5). Three GO terms were enriched in the category of cellular component, “cytoplasmic part,” “cytoplasm,” and “ribosome.” Only two GO terms, “structural constituent of ribosome” and “structural molecule activity,” were enriched in the category of molecular function.

For the Low treatment, there was one and only GO term, “translation,” in the biological process category (Figure 5). In the cellular component category, “ribosome,” “non-membrane-bounded organelle,” “intracellular non-membrane-bounded organelle,” “ribonucleoprotein complex,” “cytoplasmic part,” “cytoplasm,” and “macromolecular complex” were enriched. “Structural constituent of ribosome” and “structural molecule activity” were enriched in the molecular function category.

### KEGG Functional Annotation

**FIGURE 6:**
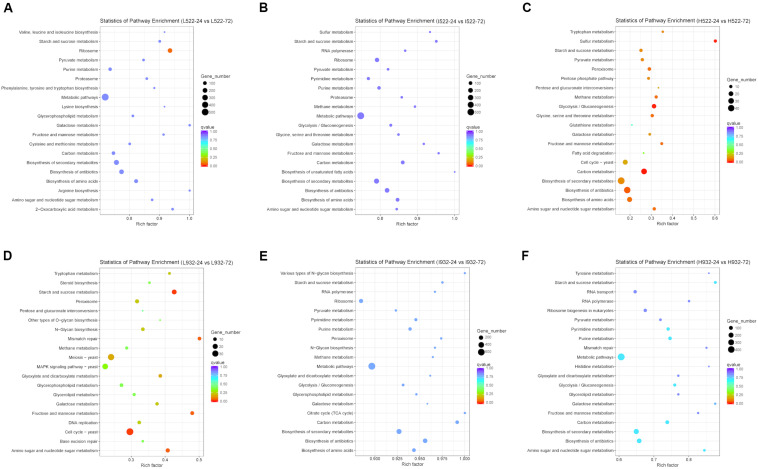
The 20 most enriched (*P* value < 0.05) Kyoto Encyclopedia of Genes and Genomes (KEGG) pathways associated with Saccharomyces cerevisiae [UCD522 **(A–C)** or UCD932 **(D–F)**] genes under three diammonium phosphate (DAP) treatments [Low (22.3 mg L^−1^) **(A,D)**, Intermediate (144.3 mg L^−1^) **(B,E)**, and High (369.3 mg L^−1^) **(C,F)**] after 24 and 72 h in a cider fermentation.

Based on our gene expression data, metabolic pathways in the KEGG database were differentially regulated between UCD522 and UCD932 significantly under three DAP treatments (Figure 6). When including DEGs from both yeast strains with all different treatments in the analysis, pathways listed with black text were significantly enriched. Those shown in blue or red were significantly enriched when only genes with higher expression at 24 or 72 h, respectively, were included in the analysis. Numbers shown in the green circles match the same pathway shown in panel A, meaning these pathways were enriched in both analyses. Pathways shown in bold remained significant following FDR correction (Supplementary Table S4).

For UCD932 (*P* value <0.05), under High treatment “Carbon metabolism,” “Purine metabolism,” “Starch and sucrose metabolism,” “Biosynthesis of secondary metabolites,” and “Metabolic pathways” were the top four enriched pathways (Figure 6). Under Intermediate treatments, “Metabolic pathways” and “Biosynthesis of antibiotics” were the top two most enriched pathways. For the Low treatment, “Cell cycle-yeast,” “Starch and sucrose metabolism,” “Fructose and mannose metabolism,” “Mismatch repair,” “Amino sugar and nucleotide sugar metabolism,” “Meiosis-yeast,” “Glyoxylate and dicarboxylate metabolism,” “Galactose metabolism,” “Peroxisome,” “N-Glycan biosynthesis,” “Tryptophan metabolism,” “DNA replication,” and “MAPK signaling pathway-yeast” were the top 13 most enriched pathways (Figure 6 and Supplementary Table S4).

To better understand metabolic pathways unique to the H_2_S-producing UCD522 strain, we excluded all UCD932 differentially regulated KEGG pathways from further analysis. For the High treatment under the UCD522 fermentation, sulfur metabolism pathways were enriched (Figure 6 and Supplementary Table S4). Among which, “Sulfur metabolism,” “Glycolysis/Gluconeogenesis,” “Amino sugar and nucleotide sugar metabolism,” “Peroxisome,” “Glycine, serine and threonine metabolism,” “Methane metabolism,” “Fructose and mannose metabolism,” “Biosynthesis of amino acids,” “Pyruvate metabolism,” “Tryptophan metabolism,” “Pentose phosphate pathway,” “Galactose metabolism,” “Cell cycle-yeast,” and “Pentose and glucuronate interconversions” were the most enriched pathways. For the Intermediate treatment, “Biosynthesis of secondary metabolites,” “Carbon metabolism,” and “Biosynthesis of amino acids” were the top three enriched pathways. Under the Low treatment, “Ribosome” was the only pathway that was enriched.

Biosynthesis of amino acids was significantly down-regulated (Figure 6 and Supplementary Table S4). Under the UCD932 fermentation, the KEGG analysis showed that “sulfur metabolism” was not enriched regardless of the DAP treatment. Under the UCD522 fermentation, when we compared the 24- and 72-h time points, 9 and 14 out of the 15 genes involved in the sulfur metabolism pathways were down-regulated for the Intermediate and High treatments, respectively. Sulfite reductase (*MET5* and *MET10*; EC 1.8.1.2), which catalyzes the direct six-electron reduction of sulfite into sulfide, was significantly up-regulated. Additionally, under both the Intermediate and High treatments, cysteine synthase (*MET17*; EC 2.5.1.47) was significantly up-regulated. Cystathionine gamma-synthase (*STR2*; EC 2.5.1.48), sulfate adenylyltransferase (*MET3*; EC 2.7.7.4), and ADP/ATP adenylyltransferase (*APA1* and *APA2*; EC: 2.7.7.53) were down-regulated under the High treatment and up-regulated under the Intermediate treatment.

## Discussion

In this study, a transcriptomics-based approach was used to examine how YAN concentration impacted H_2_S production and subsequently mRNA levels and gene expression for *S. cerevisiae* yeast strains during a cider fermentation. Specifically, by comparing a natural (UCD522) to a mutated (UCD932) yeast strain under increasing DAP concentrations, we characterized genes involved with H_2_S production within the sulfate reductase pathway. We found that the DAP treatments affected H_2_S production and fermentation kinetics. Further, the genome-wide analysis detected a large set of *S. cerevisiae* genes that were differentially expressed as a result of the DAP treatments.

Moderate DAP supplementation to 208 mg L^–1^ YAN resulted in a 2.3-fold increase in total H_2_S production, whereas higher supplementation to 433 mg L^–1^ decreased total H_2_S production to a level below the Low (86 mg L^–1^ YAN) DAP treatment. For UCD522, this curvilinear response is in contrast with the generally accepted paradigm that increasing YAN concentration reduces the H_2_S production during fermentation (Vos and Gray, 1979; Jiranek et al., 1995). In particular, the early results of Vos and Gray (1979) established the existence of a negative correlation between YAN and H_2_S production. However, in that study, the average YAN of the tested grape juice was 208 mg L^–1^, which suggests that their results were based on a YAN concentration much greater than the apple juice in our study.

We examined the gene expression profiles of the two yeast strains to understand their phenotypic diversity and gain insights into the molecular and physiological mechanisms associated with differences in their fermentation capacities. For UCD522 at the two sampling time points, we identified 3,877 (L522-24 vs L522-72), 3554 (I522-24 vs I522-72), and 439 (H522-24 vs H522-72) DEGs in the Low, Intermediate, and High DAP treatments, respectively.

After examining the DEGs using GO terms and KEGG pathways, we investigated which genes or pathways potentially contributed to the differing H_2_S production in our treatments. At 24 h, the Intermediate and High DAP treatments for UCD522 led to similar gene expression profiles that were both different from the Low DAP treatment expression. This is consistent with other studies (Jiranek et al., 1995; Spiropoulos and Bisson, 2000). However, at 72 h, the Low and Intermediate DAP treatments had similar gene expression. Additionally, the Low and Intermediate treatments released more H_2_S than the High treatment during the UCD522 fermentations.

As previously reported, H_2_S production during alcoholic fermentation largely results from enzymatic activity in the SRS pathway (Figure 2). Further, the expression of MET genes are reportedly tightly correlated with yeast growth (Patton, 2000). In our study, at 24 h for UCD522, we found that *HOM2* was negatively correlated with the YAN concentration, and *HOM3*, *MET10*, *MET2*, and *SAH1* were positively correlated with increasing YAN concentration.

*HOM2* is required for *O*-acetyl-L-homoserine synthesis, and the inability to produce this compound would be expected to result in the production of sulfide similar to the loss of *MET17* reported elsewhere. Our results are similar to those reported by Spiropoulos and Bisson (2000) in that *MET17* expression levels were not correlated with H_2_S production. Higher H_2_S production did not lead to an up-regulation of genes involved in *O*-acetyl-L-homoserine production, such as *MET2* or *HOM3*. Thus, H_2_S production is not solely due to the absence of *O*-acetyl-L-homoserine. Another explanation is that L-aspartyl-4-P is possibly an inducer of the sulfate reduction pathway or otherwise regulates sulfate reduction such that the loss of *HOM3* results in a decrease in the pathway activity and compensates for the loss of reduced sulfide incorporation. It is important to note that mRNA levels do not necessarily correlate with protein levels or protein activity, which could also explain the observed discrepancies.

At 72 h, *CYS3*, *GSH1*, and *MET10* had negative correlations with H_2_S production. This is possibly because *MET5* or *MET10* mutations block the conversion of sulfite to sulfide and therefore reduced H_2_S production (Sutherland-Stacey et al., 2008; Cordente et al., 2009). Overexpression of the cystathionine synthetase (*CYS4*) may also reduce H_2_S production by driving sulfide toward amino acid synthesis (Tezuka et al., 1992). *MET14* has been shown to limit sulfur assimilation (Donalies and Stahl, 2002), which we also found in our work (Figure 4). Additionally, mutations in *MET2* (produces *O*-acetyl-homoserine) or *SKP2* (a potential regulator of sulfur assimilation genes) increases sulfite and H_2_S production (Hansen and Kielland-brandt, 1996).

Using a preferred nitrogen source, such as DAP, for fermentations, *ECM38* expression is repressed through a mechanism involving the Gln-binding protein Ure2/GdhCR (Springael and Penninckx, 2003). Inadequate nitrogen concentration (i.e., less than 140 mg N/L) could induce *ECM38* expression, meaning that the L932-24, L932-72, I932-24, L522-24, I522-72, and L522-72 treatments were nitrogen deficient in our study (Figure 4). It is possible that this resulted in the higher expression of *MET10, HOM3*, *HOM2*, and *MET2* genes, since their activity was correlated with reduced H_2_S production (Patton, 2000; Spiropoulos et al., 2000; Linderholm et al., 2010; Huang et al., 2013).

We did not find any GO terms enriched in thiamine biosynthesis (Figure 5). Our results were not in agreement with the previous suggestion that greater H_2_S production is correlated with thiamine biosynthesis gene expression (Bartra et al., 2010). However, for UCD522 sampled at 24 h, there was a positive correlation between the DAP treatments and expression quantity of *HOM3*, *MET10*, *MET2*, and *SAH1* genes.

Mendes-Ferreira et al. (2008) reported that, in a model grape juice, MET genes involved in the formation of H_2_S were specifically down-regulated under nitrogen deficiency (YAN = 66 mg L^–1^), while supplementation of nitrogen to an initial YAN of 267 mg L^–1^ resulted in up-regulation of these genes and maximum H_2_S formation. For UCD522, 83.3% of MET genes involved in the formation of H_2_S were down-regulated under YAN 86 mg L^–1^. Unlike previous reports, only three genes (*MET1*, *MET8*, and *MET28*) were up-regulated under the Intermediate DAP treatment in our study, while the High treatment resulted in the up-regulation of half of these genes. Moreover, the Intermediate treatments entered the stationary fermentation phase between day 6 and 7, while H_2_S was released between day 3 and 4. Therefore, sulfite reductase activity was likely up-regulated during the growth phase when nitrogen was limited. The observation that low YAN juice determines a non-linear dose response for H_2_S production is particularly relevant in light of generally low nitrogen content for apples (Ma et al., 2018).

Although DAP supplementation is often used by commercial cider producers to reduce H_2_S production during fermentation, this common practice does not appear to consistently produce this result. In fact, in our study, moderate nitrogen supplementation to a low nitrogen juice was generally associated with increased H_2_S production. Given the importance of H_2_S management for the modern cider industry, our results suggest that further research should target additional mechanisms involved with H_2_S production.

Previous studies report that H_2_S formed late in fermentation could be responsible for increased residual H_2_S that affects the sensory quality of fermented beverages (Karagiannis and Lanaridis, 1999). In the UCD522 fermentation, the Low and Intermediate DAP treatments resulted in significantly greater H_2_S production compared to the High treatment. Furthermore, according to our RNA-Seq data, the expression level of three genes (*MET10*, *HOM3*, and *HOM2*) was correlated with YAN concentration, and two genes (*MET10* and *CYS3*) were correlated with greater H_2_S production. It is possible that this is the result of the up-regulated expression of *MET10*, since its activity was correlated with reduced H_2_S production (Cordente et al., 2009). Overall, it appears that H_2_S biosynthesis during cider fermentation is under a tight regulatory control by *MET10*.

The similarity in sulfur metabolism between 24 and 72 h under the Low DAP treatment using the KEGG and GO analyses was possibly due to the very low starting YAN concentration in the apple juice. By comparison, the Intermediate and High treatments started with a concentration above 140 mg YAN⋅L^–1^ and decreased to below the deficiency level before 72 h. Different nitrogen concentrations at the two time points may have led to the observed H_2_S production and metabolism findings.

In summary, we confirm a non-linear relationship between YAN and H_2_S production during cider fermentation, with the low and intermediate YAN levels having greater H_2_S production than the high YAN level. The RNA-Seq analysis allowed us to uncover a complex coordination between the genes involved with a stress response and H_2_S production. Our study demonstrates the importance of gene expression analysis in understanding yeast response to YAN concentrations and sheds light on the molecular basis of yeast physiology and H_2_S production during cider fermentation. Further investigations should be aimed at exploring the management of the nitrogen anabolic requirements in cider fermentation, as well as the effect of organic nitrogen sources, such as amino acids.

## Data Availability Statement

The datasets generated for this study can be found in NCBI SRA, accession PRJNA592382, https://www.ncbi.nlm.nih.gov/bioproject/PRJNA592382.

## Author Contributions

YS conducted the experiment, performed the RNAseq, analyzed the data, and prepared the manuscript. PG, LC, and SL assisted with data analyses and manuscript preparation. GP developed the experimental design, assisted with data analyses and interpretation, edited the manuscript for submission, and supervised YS. All authors contributed to the article and approved the submitted version.

## Conflict of Interest

The authors declare that the research was conducted in the absence of any commercial or financial relationships that could be construed as a potential conflict of interest.

## Footnotes

1 http://www.kegg.jp

2 http://www.yeastgenome.org

## Supplementary Material

FIGURE S1 The relative expression levels of selected genes compared between **(A)** quantitative reverse transcription polymerase chain reaction qRT-PCR and **(B)** RNA-Seq analysis. Relative expression levels of selected genes in the strains at first place of comparison were set to 1. Regression analysis **(C)** of the relative expression level of selected genes in UCD932 and UCD522. Each dot represents a single gene.
