# Supplementary material for: Yeast Assimilable Nitrogen Concentrations Influence Yeast Gene Expression and Hydrogen Sulfide Production During Cider Fermentation
Source: Front Microbiol. 2020 Jun 24;11:1264. doi: 10.3389/fmicb.2020.01264 (PMC7326769; doi:10.3389/fmicb.2020.01264)
Supplement: Supplementary file 5 [file Data_Sheet_1.docx]

SUPPLEMENTARY MATERIAL

Article title: Yeast Assimilable Nitrogen Concentrations Influences Yeast Gene Expression and Hydrogen Sulfide Production during Cider Fermentation

Authors: Yangbo Song^1,2^, Patrick Gibney^3^, Lailiang Cheng^2^, Shuwen Liu^1*^, and Gregory Peck^2*^

^1^College of Enology, Northwest A&F University, Yangling, Shaanxi, China

^2^School of Integrative Plant Science—Horticulture Section, Cornell University, Ithaca, NY, USA

^3^Department of Food Science, Cornell University, Ithaca, NY, USA

*** Correspondence:**

Gregory Michael Peck: [gmp32@cornell.edu](mailto:gmp32@cornell.edu)

Shuwen Liu: liushuwen@nwsuaf.edu.cn

**TABLES:**

Table S1. Differential gene expression for UCD522. There were 3,877 genes differentially expressed between L522-24 and L522-72 (1,974 DEGs were up-regulated and 1,903 DEGs were down-regulated), 3,554 genes differentially expressed between I522-24 and I522-72 (1,796 DEGs were up-regulated and 1,758 DEGs were down-regulated), whereas 439 genes were differentially expressed between H522-24 and H522-72 (239 DEGs were up-regulated and 200 DEGs were down-regulated).

Table S2. Differential gene expression for UCD932. Between 24 and 72 hours (L932-24 and L932-72) there were 826 DEGs (447 were up-regulated and 379 were down-regulated). Comparison of I932-24 and I932-72 was 4,615 DEGs (2,332 DEGs were up-regulated and 2,293 were down-regulated). For H932-24 and H932-72 there were a total of 3,020 DEGs (1,535 DEGs were up-regulated and 1,485 were down-regulated).

Table S3. Gene Ontology enrichment analysis was performed with the differentially expressed genes of six comparisons (L522-24 vs L522-72, I522-24 vs I522-72, H522-24 vs H522-72, L932-24 vs L932-72, I932-24 vs I932-72, and H932-24 vs H932-72). DEGs were assigned to one or more GO terms and categorized into 1,770 (L522-24 vs L522-72), 1,800 (I522-24 vs I522-72), 834 (H522-24 vs H522-72), 1,128 (L932-24 vs L932-72), 1,923 (I932-24 vs I932-72), and 1,664 (H932-24 vs H932-72) GO terms in the three main categories (biological process, molecular function, and cellular component).

Table S4. Kyoto Encyclopedia of Genes and Genomes (KEGG) functional annotation between UCD522 and UCD932 yeast strains under three diammonium phosphate treatments.

**FIGURES:**

Figure S1. Validation of the RNA-seq using qRT-PCR. Relative expression levels of selected genes between the methods were well correlated (r^2^ = 0.88; P ≤ 0.0001).
