# Supplementary figures and images for: Yeast Assimilable Nitrogen Concentrations Influence Yeast Gene Expression and Hydrogen Sulfide Production During Cider Fermentation
Source: Front Microbiol. 2020 Jun 24;11:1264. doi: 10.3389/fmicb.2020.01264 (PMC7326769; doi:10.3389/fmicb.2020.01264)

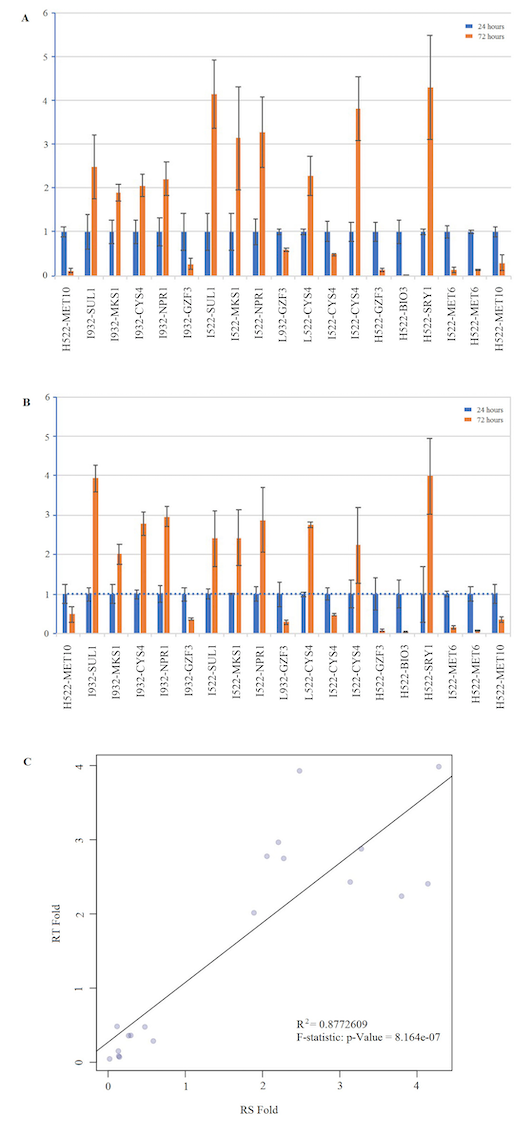

Supplement: FIGURE S1 — The relative expression levels of selected genes compared between (A) quantitative reverse transcription polymerase chain reaction qRT-PCR and (B) RNA-Seq analysis. Relative expression levels of selected genes in the strains at first place of comparison were set to 1. Regression analysis (C) of the relative expression level of selected genes in UCD932 and UCD522. Each dot represents a single gene. [file Image_1.tiff]
